# Differential Effects of Zanubrutinib and Ibrutinib on Neutrophil Antifungal Functions: Insights from Live-Cell Imaging

**DOI:** 10.1159/jin/adhag001

**Published:** 2026-08-14

**Authors:** Benoit S. Marteyn, Aymeric Silvin, Tamou Thahouly, Nathan Broussaudier, Jean-Daniel Fauny, Marina Valente Barroso, Thierry Fontaine, Kamel Laribi, David Ghez

**Affiliations:** a Université de Strasbourg, Institut de Biologie Moléculaire et Cellulaire (IBMC), CNRS UPR9002, Strasbourg, France; b Institut Pasteur, INSERM U1225, Unité de Pathogenèse des Infections Vasculaires, Paris, France; c University of Strasbourg Institute for Advanced Study (USIAS), Paris, France; d INSERM U1015, Institut Gustave Roussy, Villejuif, France; e Université de Strasbourg, Institut de Biologie Moléculaire et Cellulaire (IBMC), CNRS UPR3572 Strasbourg, France; f Institut Pasteur, Unité Biologie et Pathogénicité Fongiques, Paris Cedex 15, France; g Centre Hospitalier Le Mans, Service Hématologie Clinique, Le Mans, France; h Institut Gustave Roussy, Département d’Hématologie, Villejuif, France; i Inserm UMR, Institut Gustave Roussy, Villejuif, France

**Keywords:** Ibrutinib, Zanubrutinib, Neutrophils, Fungal infections, Parasite-host interactions

## Abstract

**Introduction:**

Bruton tyrosine kinase inhibitors (BTKi) impair anti-*Aspergillus* immune responses and increase the risk of invasive fungal infections (IFI). More selective second-generation BTKi have fewer off-target effects thus a better tolerance than ibrutinib. Though clinical data suggest the incidence of IFI is lower with second-generation BTKi, whether they have less impact on antifungal immunity remains unclear.

**Methods:**

We studied the activation phenotype and behavior of human neutrophils in vitro with fungal extracts or living *Aspergillus fumigatus* in the presence of either ibrutinib or zanubrutinib, a more selective second-generation BTKi, using various approaches, including live microscopy.

**Results:**

Both molecules inhibited the activation-induced phenotypic changes in neutrophils following exposure to hyphae. Similarly, ibrutinib and zanubrutinib impaired neutrophil-mediated inhibition of conidia germination. By contrast, live neutrophil imaging showed that neutrophils exposed to ibrutinib but not zanubrutinib kept a round morphology, suggesting a defective activation, were less mobile, and showed impaired conidia phagocytosis.

**Conclusion:**

We confirm here that ibrutinib and zanubrutinib inhibit neutrophil antifungal activity, strongly suggesting that BTK plays a central role. However, subtle differences between ibrutinib and zanubrutinib were evidenced using live microscopy, which might indicate additional off-target effects, and potentially partially explain the lower incidence of IFI with zanubrutinib; the clinical relevance remains to be determined.

## Introduction

Treatment with BTKi inhibitors (BTKi), in particular, with the first-in-class molecule ibrutinib, has been linked to an increased susceptibility to invasive fungal infections (IFI), the most frequent being invasive aspergillosis, a life-threatening disease with a high mortality rate [1–4]. This was unexpected since X-linked agammaglobulinemia, a primary immune deficiency caused by inactivating BTK mutations, is not associated with an increased susceptibility to fungi [5]. Initially described as a critical component of the B-cell receptor signaling pathway, the role of BTK extends beyond B cells. In particular, BTK is expressed by neutrophils where it participates to neutrophil development and activation [6–8].

We and others found that neutrophils exposed to BTKi in vitro and in vivo displayed multiple functional defects, such as impaired activation, inhibition of ROS production and defective phagocytosis and killing of *Aspergillus fumigatus* [9–14]. In neutrophils, BTK is activated downstream of Dectin-1, TLR2, and FcγRIIA/IIIB after fungal exposure [12]. BTK activation leads to ROS production, primary granule release, and hyphal damage, all processes that are abrogated upon ibrutinib exposure.

If all molecules of the BTKi family strongly inhibit the kinase activity of BTK, they have distinct pharmacological properties. In particular, they were designed to increase their affinity and specificity to BTK and thus have a narrower kinase inhibition profile. This translates into fewer off-target effects that explain their better tolerance in comparison to ibrutinib, in particular, in terms of cardiotoxicity, a well-known complication of BTKi. Differences in their effect on the immune response have also been described. For example, ibrutinib, but not acalabrutinib, interferes with FcγR signaling and inhibits antibody-dependent cellular phagocytosis and cytotoxicity [15, 16]. This property explains why the addition of anti-CD20 monoclonal antibodies, a mainstay of the treatment of B-cell malignancies, to ibrutinib demonstrate no clinical benefit. Whether off-target effects play a role in the inhibition of immune responses against fungi remain an outstanding question.

The majority of studies on fungal infections have focused on ibrutinib. Data on second-generation BTKi such as acalabrutinib and zanubrutinib are limited to anecdotal cases [11, 17] and the results of clinical trials, which seem to indicate that IFI are less frequent with newer BTKi. However, it should be kept in mind that ibrutinib, during its clinical development, was given to heavily pretreated, hence often severely immunocompromised, patients. This may have biased the comparison inasmuch as it is well recognized that ibrutinib by itself is usually not sufficient for IFI to develop but requires additional factors such as neutropenia or concomitant corticosteroid therapy. Therefore, the fact that most patients now receiving BTKi were never exposed to immunochemotherapy could by itself explain the decrease of BTKi-associated IFI in recent years. Whether the susceptibility to fungi differs between commercially available BTKi is thus not completely clear. In this work, we compared the effect of ibrutinib and zanubrutinib, a more selective second-generation BTKi, on neutrophil antifungal activity, using *A. fumigatus* as the fungus model.

## Methods

### Human Blood Collection and Neutrophil Purification

All participants gave written, informed consent in accordance with the Declaration of Helsinki principles. Peripheral human blood was collected from healthy volunteers at the Etablissement Français du Sang (EFS) de Strasbourg (authorization no. ALC/PIL/DIR/AJR/FO/606). As described previously [18, 19], neutrophils were isolated from 8 mL of human whole blood in citrate tubes collected the same day at the EFS using the MACSxpress Whole Blood Neutrophil Isolation Kit, human (Miltenyi Biotec). The remaining red blood cells were removed using the MicroBeads human CD235a (Glycophorin A) kit (Miltenyi Biotec). Purified neutrophils were centrifuged at 300×*g* for 10 min and were subsequently resuspended in 1 mL Roswell Park Memorial Institute (RPMI) medium (32404014, Gibco™) supplemented with 10 mm HEPES + 10% (v/v) fetal bovine serum. When indicated, neutrophils were fixed in 3.3% (w/v) paraformaldehyde (PFA) for immunostaining.

### *A. fumigatus* Culture

*A. fumigatus* strain S1 pDsRed Dal 521 (kindly supplied by Dr. Thierry Fontaine, Institut Pasteur) was cultured at room temperature on Malt agar (Sigma-Aldrich). Conidia were separated from hyphae by collecting 14-day cultures in 5 mL Tween 0.05% (v/v) and filtered onto 40-μm cell strainer (Fisher Scientific). Eluted conidia were centrifuged at 1,200×*g* for 15 min and were resuspended in 1 mL RPMI medium supplemented with 10 mm HEPES. Hyphae were collected onto the filter and resuspended in the same media.

When indicated, germination of purified conidia was performed at 37°C during 3 h in RPMI medium supplemented with 10 mm HEPES, using 10^6^ conidia/well in 24-well plates. To study the impact of ibrutinib and zanubrutinib on *A. fumigatus* conidia germination, the experiment was performed for 1 and 6 h in the presence of 1 μm ibrutinib or zanubrutinib. For immunofluorescence imaging, conidia were fixed in 3.3% (w/v) PFA for 30 min at 37°C and further stored in phosphate buffered saline (PBS) at 4°C prior immunostaining.

*A. fumigatus* cell wall extracts were also produced and supplied by Dr. Thierry Fontaine (Institut Pasteur). The alkali-insoluble fraction was obtained from *A. fumigatus* mycelium cell wall [20]. This fraction was treated by periodate oxidation to eliminate the galactomannan; it contains only a mixture of chitin and β-1,3-glucan, as previously demonstrated [20, 21].

### Drugs, Antibodies and Reagents

Ibrutinib is commercially available (ref. FLUH99CD1F73, Merck) and zanubrutinib was supplied by BeiGene in the frame of an ISR study. Both drugs were initially resuspended in DMSO at 1 mm.

Commercial conjugated antibodies used for flow cytometry and spectral flow cytometry analysis are listed in Table S1 and were all used at a dilution 1:100, as recommended. Additional antibodies and fluorescent markers used for immunofluorescent imaging are listed below. Myelotracker-Cy5/Cy3 (Bertin Technologies), a specific lactoferrin peptide marker, was used at a final concentration of 1 μg/mL [20]; the anti-β-glucan (β-1,3-glucan) mouse monoclonal antibody (5H5) (kindly supplied by Dr. Vishu Kumar Aimanianda Bopaiah, Institut Pasteur) was used at a dilution 1:2,000 [22]; Dectin mouse anti-human (ref. 15205959, Thermo Fisher Scientific) was used at a dilution 1:500; Phalloidin Alexa Fluor 555 (Thermo Fisher Scientific, ref. A34055) was used at a dilution 1:1,000; Alexa Fluor Plus 488 mouse antibody (Thermo Fischer Scientific, ref. A32723) was used at a dilution 1:1,000; and Alexa Fluor Plus 488 rabbit (Thermo Fischer Scientific, ref. A32731) was used at a dilution 1:1,000. DAPI (4′,6-diamidino-2-phenylindole) (Thermo Fischer Scientific, ref. D1306) was used at a final concentration of 1 μg/mL.

### Neutrophil Infection and Activation

To assess the impact of ibrutinib or zanubrutinib on neutrophil survival and antifungal functions, purified cells were cultured in 24-well plates or Ibidi™ 8-well plates (Thermo Fisher Scientific™) containing, respectively, 1 mL or 300 μL RPMI 1640 medium + 10% (v/v) fetal calf serum + 10 mm HEPES. Drug concentrations ranged between 1 μm and 50 μm, as indicated. DMSO was used as a control (vehicle).

When indicated, neutrophils were infected with purified conidia at an MOI = 1. Infected neutrophils were subsequently centrifuged at 300×*g* for 10 min and incubated at 37°C during the indicated infection time. For immunostaining, samples were fixed in 3.3% (w/v) PFA; for flow cytometry analyses, cells were resuspended in PBS + 2 mm EDTA; for spectral flow cytometry, cells were resuspended in a Cytodelics fixative solution.

The impact of ibrutinib or zanubrutinib on the capacity of neutrophils to release neutrophil extracellular traps (NETs) [23] or proteoglycofili (PGF) [24] was assessed by culturing neutrophils onto coverslips in 24-well plates under normoxia in the presence of 10 nm PMA (phorbol-12-myristate-13-acetate) (Merck) or under anoxia for 4 h (using a Whitley A20 anoxic workstation, Don Whitley Scientific), respectively. Samples were fixed with a Carnoy’s fixative solution prior immunolabeling. In all tested conditions, experiments were performed on at least three independent samples.

### Cell Immunolabeling and Imaging

For immunofluorescence, *A. fumigatus* (conidia or hyphae) and neutrophils were first fixe d in PFA 3.3% (w/v). Samples were washed three times in PBS supplemented with 0.1% (v/v) Triton X-100 and stained for 1 h at room temperature with the primary antibodies listed above. Samples were subsequently washed three times in PBS supplemented with 0.1% (v/v) Triton X-100 prior incubation with the secondary antibodies listed above or fluorescent dyes (Myelotracker, DAPI). Samples were subsequently washed three times in PBS supplemented with 0.1% (v/v) Triton X-100 then in PBS and subsequently in H_2_O and mounted with ProLong Gold^®^ (Invitrogen) and imaged using a laser-scanning TCS SP8 confocal microscope (Leica).

### Flow Cytometry Analysis

Neutrophil viability and activation were assessed by flow cytometry using SYTOX Green (Thermo Fisher Scientific™) and conjugated antibodies targeting specific markers (CD62L, CD11b, CD54), using 2.10^5^ neutrophils per condition. Cells were washed and resuspended in 350 μL of PBS + 2 mm EDTA and further incubated with marker on ice for 15 min. Experiments were performed on three independent samples. Samples were analyzed using a acquired using BD FACSCanto and BD FACSCelesta. Data analysis was performed using the BD FACSDiva software.

### Spectral Flow Cytometry

For spectral flow cytometry, neutrophils incubated or not with ibrutinib or zanubrutinib (1 μm) were infected with *A. fumigatus* conidia or hyphae (separated from the same culture batch) at MOI 1 or DMSO for 3 h at 37°C in RPMI 1640 medium supplemented with 10 mm HEPES and 10% (v/v) fetal calf serum. Cells were collected by centrifugation at 300×g for 10 min and resuspended in a mixture of 200 μL RPMI and 200 μL of Cytodelics stabilizer solution. Samples were incubated at room temperature for 10 min and subsequently frozen at −80°C.

For assays, 200,000 neutrophils were analyzed per condition. Samples were secondarily thawed in a water bath set to +37°C. Cells were fixed at a ratio 1:1 with fixation buffer (Cytodelics, ratio 1:1) and incubated for 10 min at room temperature. Red blood cells were lysed by addition of 2 mL of lysis buffer (Cytodelics, ratio 1:4) at room temperature for 10 min. Cells were washed with 2 mL of wash buffer (Cytodelics, ratio 1:5). Cells were resuspended in 100 μL extracellular antibody cocktail and incubated at 4°C for 20 min. Antibodies listed above were diluted in blocking buffer (FACS buffer + 1% (v/v) rat serum + 1% (v/v) mouse serum). All antibodies were used at 1/200 dilution and applied at 100 μL per cell suspension. Cells were incubated for 15 min with antibodies, mixing at 4°C, and then washed with 1 mL of FACS buffer, centrifuged at 600×g, for 4 min at 4°C, and resuspended in 200 μL of FACS buffer. All antibodies are listed in Table S1. Samples were acquired on Cytek Aurora flow cytometer (Cytek Biosciences). FCS files were exported and analyzed using FlowJo software. Marker expression values were transformed using the auto-logicle transformation function from the flowCore R package. Uniform Manifold Approximation and Projection (UMAP) were carried out using all markers (flow cytometry) or significant PCs (based on Seurat analysis for scRNa-seq data). UMAP was run using 15 nearest neighbors (nn), a min_dist of 0.001–0.2 and Euclidean distance [25].

### Live Microscopy

Neutrophil integrity, dynamics, and phagocytic capacity were assessed by live microscopy using an EVOS M7000 microscope (Thermo Fisher Scientific™) equipped with an incubation chamber (5% CO_2_, 70% humidity, 37°C) and a 20× objective. Cells were cultured in Ibidi™ 8-well plates (Thermo Fisher Scientific™). Then, 200,000 neutrophils were stimulated or not stimulated with *A. fumigatus* extracts or purified conidia (MOI 1) as indicated. Bright-field images were acquired every 30 s for 4 h in all tested conditions. Experiments were performed on three independent samples. Image analysis was performed with Fiji software to produce online supplementary Movies 1–12 (for all online suppl. material, see https://doi.org/10.1159/jin/adhag001) and Qupath software for quantitative analyses.

### Statistical Analysis

In all experiments, *n* indicates the number of biological replicates. All statistical analyses were performed using GraphPad Prism 10 software. Comparisons with three groups or more were supported by one-way ANOVA tests followed by a post hoc Tukey’s test. When *p* values for the significance of pairwise comparisons were calculated and displayed on figures, we used the following convention: not significant (ns; *p* ≥ 0.05); *0.01 ≤ *p* < 0.05; **0.001 ≤ *p* < 0.01; ***0.0001 ≤ *p* < 0.001; ****0.00001 ≤ *p* < 0.0001.

## Results

### Effects of Ibrutinib and Zanubrutinib on Neutrophil Viability and Motility

The aim of this study was to evaluate the impact of ibrutinib and zanubrutinib on neutrophil antifungal activity in a comparative study. As a prerequisite, the respective sub-toxic doses of these drugs on neutrophils in vitro was first defined*.* Neutrophils were exposed to concentrations ranging from 1 μm to 50 μm over a short time-period (1 h). Neither effect on neutrophil morphology (round shape and multilobulated nucleus) (Fig. 1a) nor toxicity was observed at a concentration of 1 μm (Fig. 1b, *p* ≥ 0.05). At higher doses (≥5 μm), both ibrutinib and zanubrutinib induced cell death as assessed by flow cytometry (Fig. 1b, *p* < 0.05 and *p* < 0.01). These results led us to use 1 μm in our experiments, which corresponds to clinically achievable concentrations [26].

**Fig. 1.**
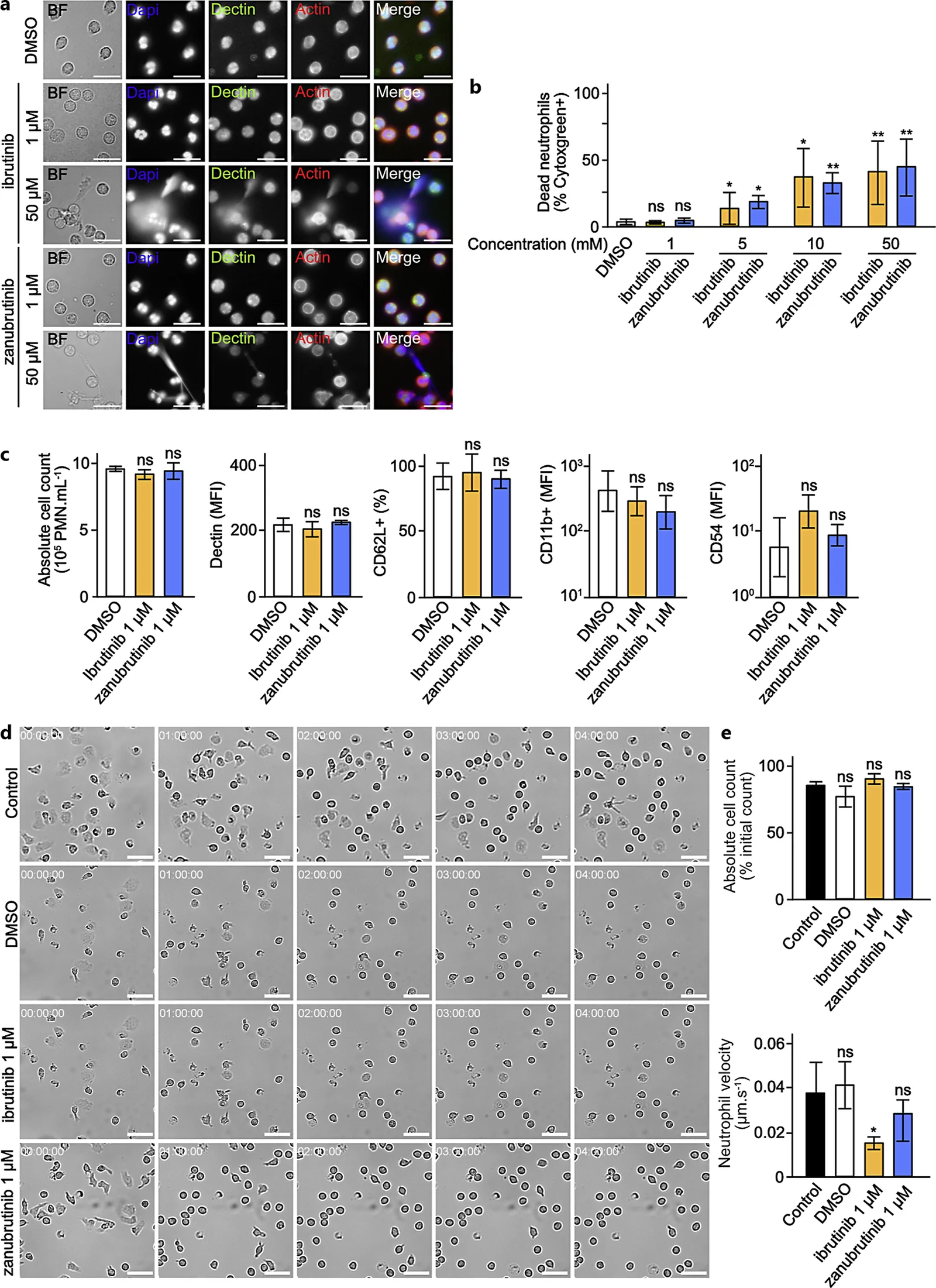
The impact of zanubrutinib on neutrophil viability and activation is similar to ibrutinib but less pronounced on neutrophil motility. **a** Human neutrophils were incubated with indicated concentrations of ibrutinib or zanubrutinib, or 5% (v/v) DMSO, in RPMI 1640 medium supplemented with 10 mm HEPES and 10% (v/v) FCS for 1 h at 37°C. Neutrophils were fixed and labeled with specific antibodies against Dectin-1 (green), DAPI (DNA, blue) and Phalloidin-Alexa 568 (actin, red). Bars, 50 μm. **b** Impact of ibrutinib and zanubrutinib at indicated concentrations on neutrophil viability, measured by flow cytometry using SYTOX Green (*n* = 3 experiments, mean ± SD). Statistical analysis performed with a one-way ANOVA with Tukey’s test: “ns” indicates *p* ≥ 0.05, * indicates *p* < 0.05, and ** indicates *p* < 0.01. **c** Impact of 1 μm ibrutinib or zanubrutinib or 0.1% (v/v) DMSO on neutrophil integrity and activation (Dectin, CD62L, CD11b, CD64) measured by flow cytometry (*n* = 3 experiments, mean ± SD). Statistical analysis performed with one-way ANOVA with Tukey’s test: “ns” indicates *p* ≥ 0.05. **d–e** Impact of ibrutinib and zanubrutinib on neutrophil dynamics, assessed by live-microscopy (pictures extracted from Movies 01–04, at indicated timepoints). Bars, 50 μm. **e** Absolute neutrophil cell count (% initial count) and velocity (μm.s^-1^) were determined between t = 0 and t = 4 h using Qupath (*n* = 3 experiments [>100 neutrophils per condition], mean ± SD). Statistical analysis performed with a one-way ANOVA with Tukey’s test: “ns” indicates *p* ≥ 0.05 and * indicates *p* < 0.05. FCS, fetal calf serum.

We further aimed at comparing the impact of ibrutinib and zanubrutinib on neutrophil activation. At 1 μm, ibrutinib and zanubrutinib had no effect on cell surface level of activation markers Dectin-1, CD62L, CD11b, and CD54 as compared to DMSO (Fig. 1c, *p* ≥ 0.05). It is well described that upon activation, neutrophils may ultimately release NETs [21] or PGF [24]. It was first confirmed that ibrutinib and zanubrutinib did not induce NET formation at 1 μm (Fig. 1a), consistent with the absence of cell death induction (Fig. 1b). Conversely, it was demonstrated that ibrutinib and zanubrutinib do not interfere with neutrophils’ ability to form NETs or PGF upon PMA stimulation under atmospheric (21% O_2_) and anoxic (0% O_2_) conditions (Online suppl. Fig. S1a, b).

The potential impact of ibrutinib and zanubrutinib on neutrophil dynamics was next questioned. Neutrophil motility was analyzed during 4 h under basal conditions in the presence of ibrutinib or zanubrutinib by live microscopy. Neutrophil adhered to the glass-bottom device and were spontaneously motile in these culture conditions (Fig. 1d-e and online suppl. Movies 1–4). Activation of neutrophils is associated with a cytoskeletal reorganization that leads to morphological changes. Most ibrutinib-exposed neutrophils kept a round shape, whereas elongated neutrophils, characteristic of activation, were observed in controls, DMSO, or after zanubrutinib exposure (Fig. 1d and online suppl. Movies 1–4). Neutrophil velocity was significantly reduced with ibrutinib (Fig. 1e *p* < 0.05 and online suppl. Movie 3) in comparison with zanubrutinib, DMSO, or control (Fig. 1d, e and online suppl. Movies 1, 2, 4). No associated modulation of neutrophil viability was observed, based on total cell count (Fig. 1e *p* ≥ 0.05). These results suggested that ibrutinib had a more pronounced inhibitory effect on neutrophil activation and mobility than zanubrutinib.

### Comparative Analysis of Neutrophil Phenotypical Modifications Induced by Ibrutinib and Zanubrutinib

To evaluate the impact of ibrutinib and zanubrutinib on neutrophil activation pathways, a comparative analysis of neutrophil phenotypical modification was performed after drug exposure, using spectral cytometry phenotyping with 21 neutrophil markers (Fig. 2a). To establish if phenotypic changes were influenced by ibrutinib or zanubrutinib, a non-supervised analysis was performed using UMAP reduction of dimension (Fig. 2b). The unsupervised analysis allowed to observe phenotypic change on neutrophils in response to hyphae in the absence of drugs compared to conidia or DMSO or control condition (online supplementary Fig. S2a).

**Fig. 2.**
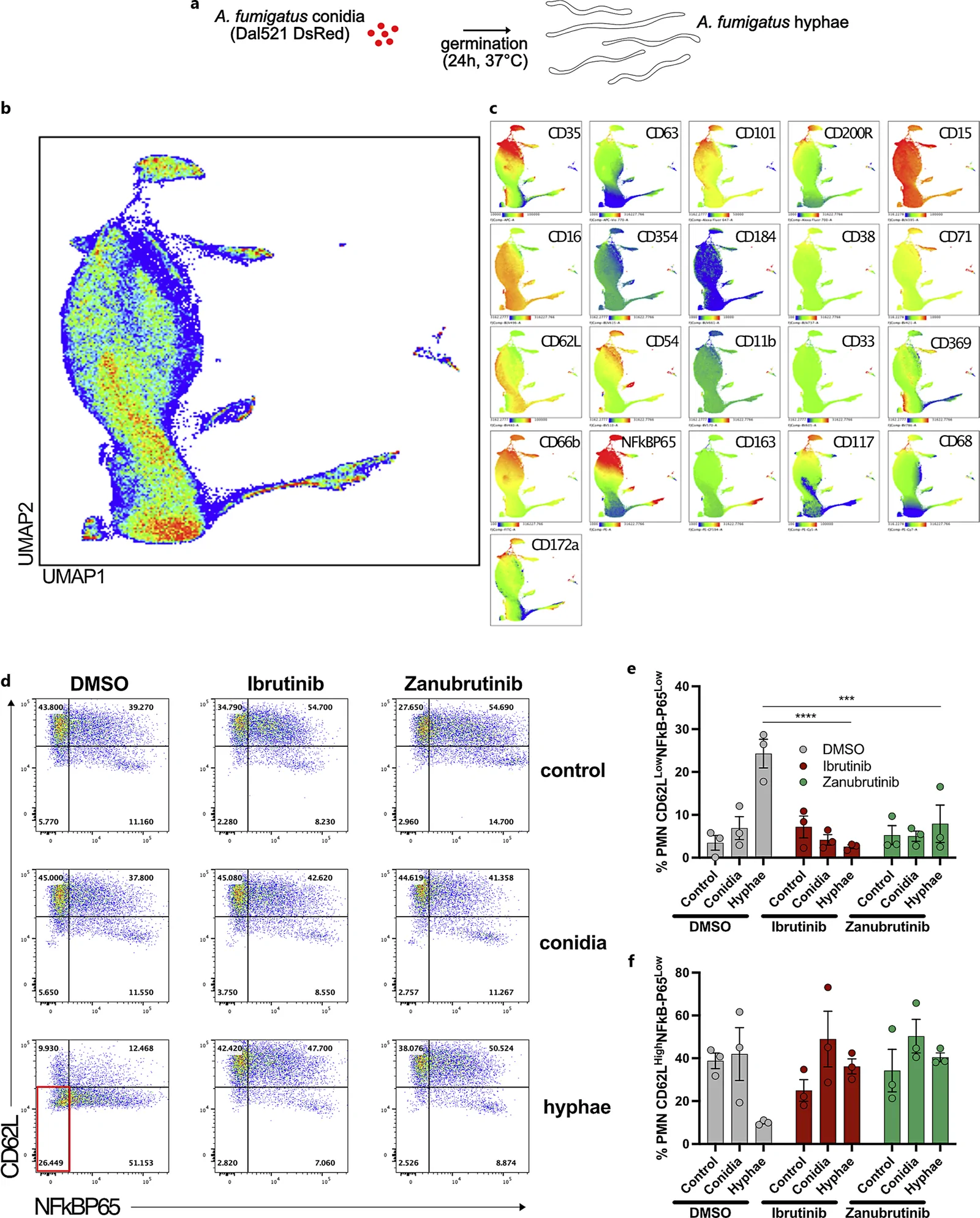
Comparative analysis of neutrophil phenotypical modifications induced by ibrutinib and zanubrutinib. **a** Experimental scheme allowing neutrophil immunophenotyping after incubation with or without conidia (MOI 1:1) or hyphae in the presence of 1 μm ibrutinib, 1 μm zanubrutinib, or 0.1% (v/v) DMSO. **b** UMAP of neutrophils from all conditions (density plot), color scale represents the density of cells (blue = low density, red = high density). **c** Expression heatmaps for each parameter measured by spectral flow cytometry (Aurora), 21 markers in total. See also Fig. S2. **d–e** Level of neutrophil CD62L and NFkBP65 markers in each condition (MOI 1:1, *n* = 3 experiments). **f** Proportion of the CD62L^Low^NFkBP65^High^ neutrophil population in different conditions described in (**e**); mean ± SD (*n* = 3 experiments). Statistical analysis performed with a one-way ANOVA with Tukey’s test: **** indicates *p* < 0.0001 and *** indicates *p* < 0.001.

As expected, exposure to hyphae was associated with a decrease of CD62L, a hallmark of neutrophil activation (Fig. 2d). CD62L shedding was inhibited by ibrutinib and zanubrutinib to a similar level (Fig. 2d-e, *p* < 0.01). Hyphae also induced CD16 downregulation, with a shift from naïve CD16^High^CD101^High^ neutrophils to a CD16^Low^CD101^Low^ population (online suppl. Fig. S2 and S4). This population was not detected in the presence of ibrutinib or zanubrutinib. Hyphae tended to increase the levels of CD66b (online suppl. Fig. S2A, B and S3A, B) and CD71 (online suppl. Fig. S5a-b), which were abrogated after exposure to both BTKi. Thus, these results confirmed that both ibrutinib and zanubrutinib blocked neutrophil activation-associated phenotypic changes in neutrophils, with no significant difference between the two drugs.

### Effect of Ibrutinib and Zanubrutinib on Neutrophils’ Ability to Phagocytize *A. fumigatus* Conidia

Conidia phagocytosis before germination represents one of the first line of defense against *A. fumigatus*. As neutrophil dynamics was differentially modulated by ibrutinib and zanubrutinib (Fig. 1d, e), their impact was further studied upon *A. fumigatus* infection using a similar approach. Neutrophils were cocultured with viable conidia in the presence of ibrutinib and zanubrutinib to quantify their impact on conidia phagocytosis using live microscopy. Neutrophils efficiently and rapidly phagocytized *A. fumigatus* conidia in control and DMSO conditions (Fig. 3a, b, online suppl. Movies 5 and 8). Ibrutinib led to reduced neutrophil velocity and decreased conidia uptake (Fig. 3a, b, *p* < 0.05 and *p* < 0.001, online suppl. Movies 5–8), whereas zanubrutinib had no significant effect, compared to control (Fig. 3a, b, *p* ≥ 0.05 and online suppl. Movies 6 and 7). Analysis of conidia internalization by fluorescent microscopy confirmed defective conidia uptake by neutrophils exposed to ibrutinib (Fig. 3c), which may also be associated with a reduced velocity.

**Fig. 3.**
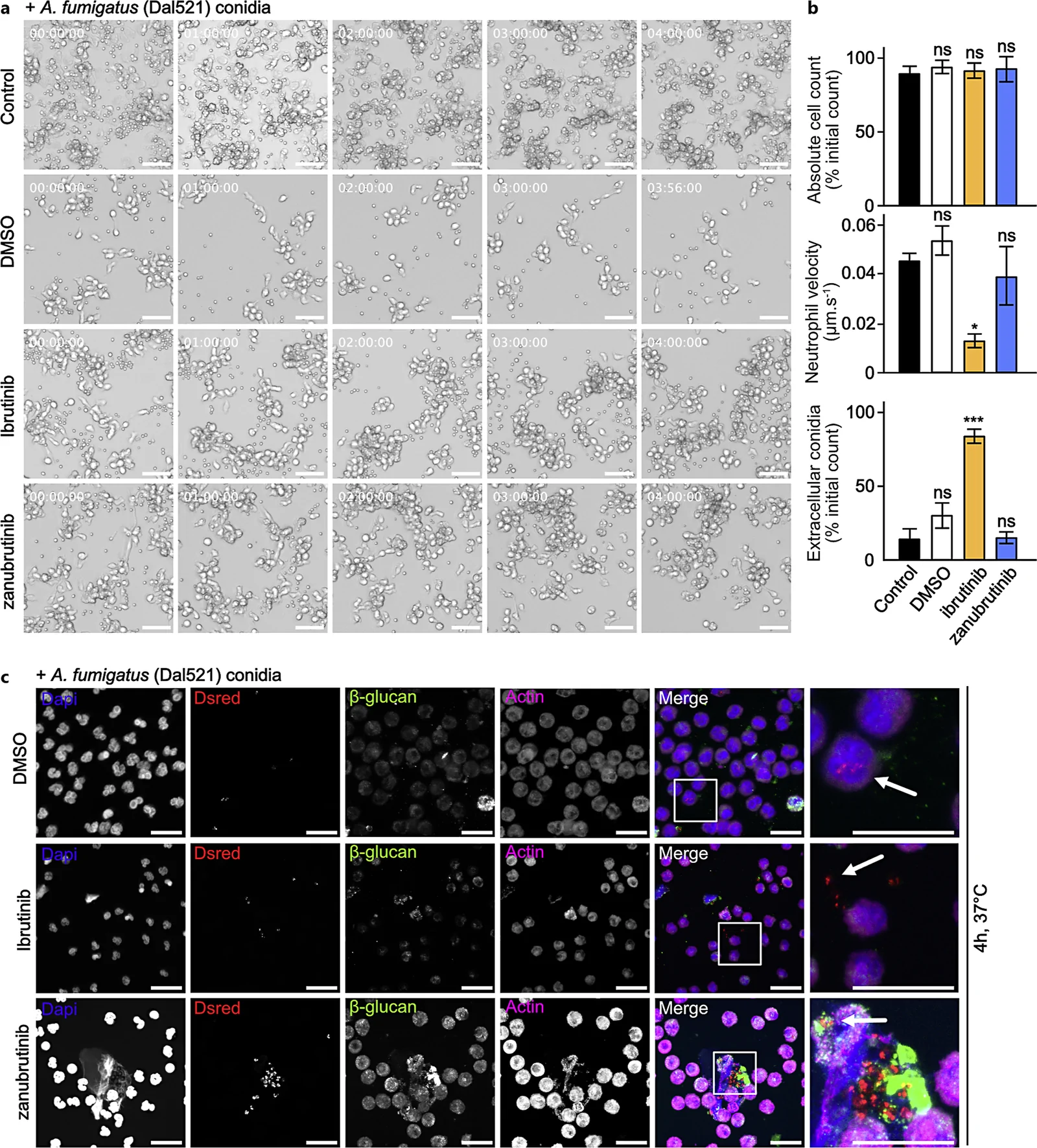
Effect of Ibrutinib and zanubrutinib on *A. fumigatus* conidia phagocytosis. **a** Neutrophils were incubated with *A. fumigatus* (Dal521) conidia (MOI 1) in the presence of 1 μm ibrutinib or zanubrutinib or DMSO during 4 h (pictures extracted from online suppl. Movies 05–08, at indicated timepoints). Bars, 50 μm. **b** Absolute neutrophil cell count (% initial count) and velocity (μm.s^-1^) were determined between t = 0 and t = 4 h using Qupath; extracellular conidia were scored at t = 0 and t = 4 h to assess neutrophil phagocytosis efficiency (*n* = 3 experiments [>100 neutrophils per condition], mean ± SD). Statistical analysis performed with a one-way ANOVA with Tukey’s test: “ns” indicates *p* ≥ 0.05, * indicates *p* < 0.05, and *** indicates *p* < 0.001. **c***A. fumigatus* conidia (Dal521, DsRed, β-glucan [green]) phagocytosis by neutrophils in the presence of 1 μm ibrutinib or zanubrutinib was assessed by immunofluorescence after 4 h of infection. DAPI (DNA, blue) and Phalloidin-Alexa 568 (actin, red). Bars, 50 μm. Arrows indicate extracellular or intracellular conidia.

### Effects of Ibrutinib and Zanubrutinib on Neutrophil-Mediated Inhibition of Conidia Germination and Recognition of Fungal Cell Wall

The impact of ibrutinib and zanubrutinib on neutrophil antifungal activity was further studied on *A. fumigatus* germinated conidia. First, it was confirmed that ibrutinib and zanubrutinib had no impact on *A. fumigatus* germination, as evidenced by the unmasking of β-glucan on the fungal cell wall (online suppl. Fig. S6a–c, *p* < 0.01). One strategy for neutrophils in controlling *A. fumigatus* infection relies on the inhibition of conidia germination. It was first determined that conidia germination was blocked by neutrophils at a ratio of 100 neutrophils per conidia (PMN:conidia ratio), not at ratios of 1 or 10 (Fig. 4a). At this ratio, we found that both ibrutinib and zanubrutinib (1 μm) abolished the capacity of neutrophils to block *A. fumigatus* germination as shown by the rapid growth of β-glucan-positive hyphae (Fig. 4b).

**Fig. 4.**
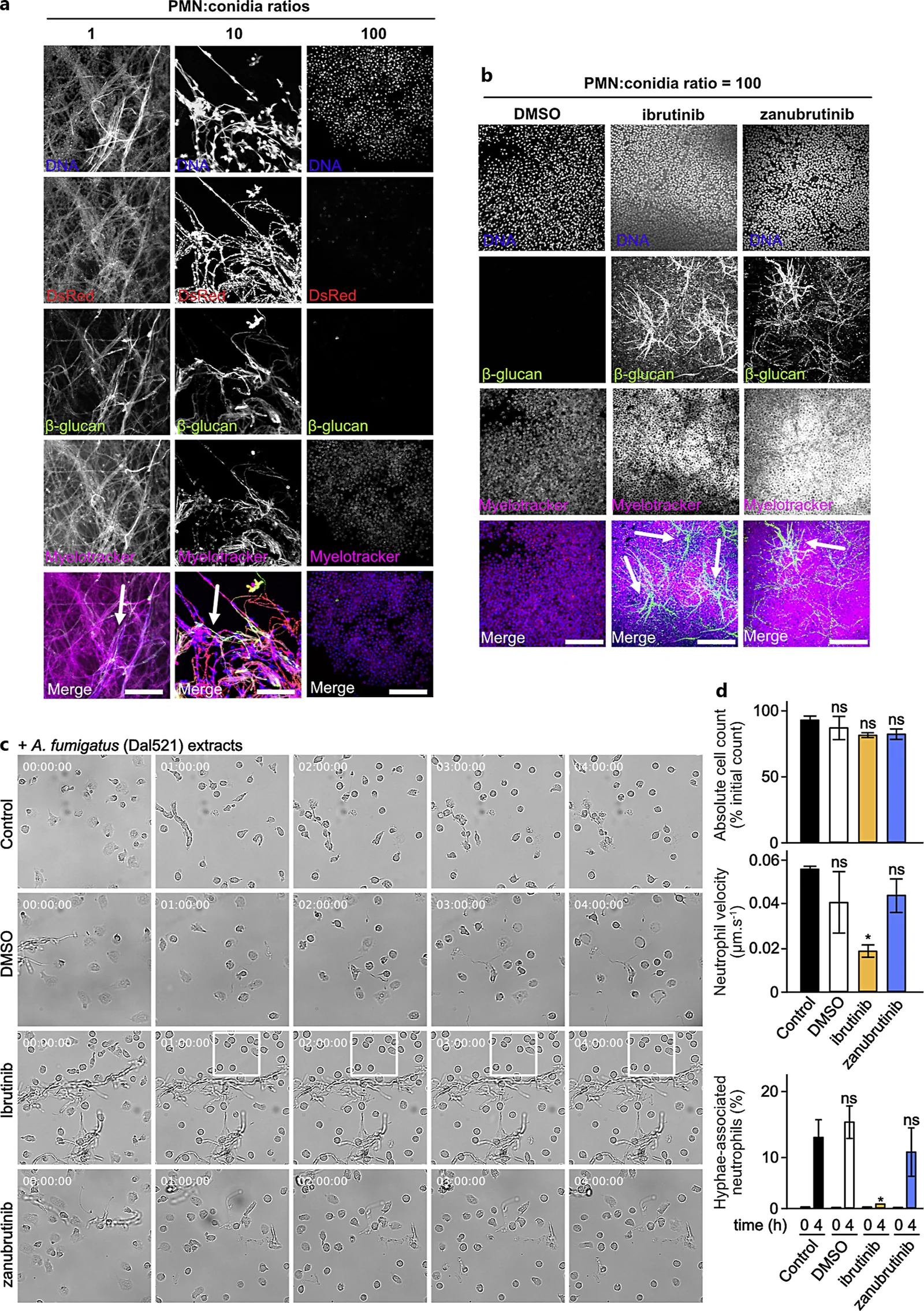
Effects of ibrutinib and zanubrutinib on neutrophil-mediated inhibition of conidia germination and recognition of fungal cell wall. **a***A. fumigatus* conidia (Dal521, DsRed) germination was induced during up to 6-h culture at 37°C then cocultured with neutrophils at the conidia/neutrophil indicated ratios. For immunodetection of cell-surface β-glucan (green) production, DNA was stained with DAPI. Bars, 50 μm. **b** Germinating *A. fumigatus* conidia were cocultured with neutrophils at a neutrophil/conidia ratio = 100 in the presence of ibrutinib or zanubrutinib. Formation of β-glucan-positive hyphae was analyzed by fluorescence microscopy. **c** Neutrophils were incubated with *A. fumigatus* (Dal521) extracts in the presence of 1 μm ibrutinib or zanubrutinib or DMSO during 4 h (pictures extracted from online suppl. Movies 09–12, at indicated timepoints). Illustration of nonreactive neutrophils upon *A. fumigatus* extracts in the presence of ibrutinib are shown in white rectangles. Bars, 50 μm. **d** Absolute neutrophil cell count (% initial count) and velocity (μm.s^−1^) were determined between t = 0 and t = 4 h using Qupath; the percentage of total neutrophils associated to *A. fumigatus* extracts was scored at 4 h (*n* = 3 experiments [>100 neutrophils per condition], mean ± SD). Statistical analysis was performed with one-way ANOVA with Tukey’s test, “ns” indicates *p* ≥ 0.05 and * indicates *p* < 0.05.

As no differential effect between ibrutinib and zanubrutinib was observed on the control of conidia germination by neutrophils, it was hypothesized that they may interfere differentially with the neutrophil dynamics in the presence of germinated *A. fumigatus*. The behavior of neutrophils stimulated with *A. fumigatus* (Dal521) extracts in the presence of ibrutinib and zanubrutinib (1 μm) was further evaluated by live microscopy. In control and DMSO conditions, stimulation of neutrophils by *A. fumigatus* extracts was associated with morphological changes and an increased velocity of neutrophils, which moved toward and interacted with *A. fumigatus* extracts. After exposure to ibrutinib, neutrophils kept their round shape and had a reduced velocity, whereas no significant inhibitory effect was observed with zanubrutinib (Fig. 4c, d, *p* < 0.05 vs *p* ≥ 0.05 and online suppl. Movies 9–12). Similarly, ibrutinib decreased the interactions between neutrophils and hyphae extracts, whereas interactions were preserved with zanubrutinib (Fig. 4c-d, *p* < 0.05 vs *p* ≥ 0.05 and online suppl. Movies 9–12). These results are consistent with our previous experiments in basal conditions showing ibrutinib impaired neutrophil activation-induced morphological changes and motility.

## Discussion

Multiple neutrophil defects have been described with not only ibrutinib but also second-generation BTKi such as acalabrutinib, tolebrutinib, evobrutinib, and fenebrutinib [10, 11, 13, 14]. These include reduced neutrophil activation as evidenced by the absence of phenotypic morphologic changes, profoundly impaired ROS production, and defective inhibition of conidia germination, whereas the effect of BTKi on neutrophil mobility was variable.

Here, we compared the effects of ibrutinib and zanubrutinib on neutrophil antifungal effector functions. Phenotypic changes such as CD62L and CD16 decrease and upregulation of CD66b and CD71 following exposure to hyphae were similarly abrogated in neutrophils cultured with ibrutinib and zanubrutinib. Zanubrutinib also impaired the neutrophil-mediated inhibition of conidia germination, a phenomenon that has already been observed with other BTKi ibrutinib, acalabrutinib, and spebrutinib [9, 10]. Multiple neutrophil defects have also been described with second-generation BTKi such as acalabrutinib, tolebrutinib, evobrutinib, and fenebrutinib [10, 11, 13, 14]. Thus, we did not observe any first significant differences between zanubrutinib, ibrutinib and other BTKi. This is consistent with the results of a recent study on acalabrutinib and zanubrutinib, which showed that both molecules similarly hindered neutrophils’ oxidative burst, swarming, and phagocytosis [13]. This accumulating evidence seem to demonstrate that all BTKi, irrespective of their generation, may be considered a risk factor of fungal infection complications in chronic lymphocytic leukemia (CLL) patients [27, 28].

When we studied the impact of ibrutinib and zanubrutinib on neutrophil morphology and behavior using live microscopy, we unraveled several subtle differences. In contrast to zanubrutinib, ibrutinib-exposed neutrophils kept a round shape, were less mobile, and displayed a decreased capacity to recognize and interact with fungal cell wall components. Interestingly, other BTKi tolebrutinib, evobrutinib, and fenebrutinib have also been shown to similarly inhibit neutrophils’ activation-induced shape changes and migratory capacity [14]. Similarly, ibrutinib impaired the recruitment and swarm formation of neutrophils cultured with *Aspergillus* spores, which could be restored by TNF-alpha [13], and acalabrutinib significantly reduced swarm formation in response to candida hyphae though to a lesser extent than with SYK inhibitors [29]. Collectively, these results suggest that zanubrutinib might have a lesser effect than other BTKi on neutrophil dynamics. Further experiments will be required to define the molecular bases of this phenotype from the cytoskeleton dynamics to cytosolic Ca^2+^ level, to ROS production [30].

Recruitment of neutrophils, release of oxygen radicals, and phagocytosis of conidia represent the initial host-defense mechanisms preventing the development of hyphae and subsequent invasion of adjacent healthy tissues [31]. Though both molecules similarly impaired the ability to block conidia germination, potentially through the strong inhibition of ROS, a constant effect of all BTKi, other experiments using live cell imaging showed that only ibrutinib significantly impaired conidia phagocytosis. Interestingly, conidia recognition and uptake are mediated by the integrin/complement receptor CR3, whose signaling is BTK-independent [30].

Thus, one hypothesis to explain the smaller incidence of IFI reported with zanubrutinib might be its lower inhibitory effect on phagocytosis compared to ibrutinib. Still, when increasing the conidia:neutrophil ratio, both ibrutinib and zanubrutinib abolish neutrophil’s capacity to block *A. fumigatus* germination.

Hypotheses to explain the slightly smaller incidence of IFI reported with zanubrutinib might thus be its lower inhibitory effect on phagocytosis and its lesser effect on neutrophil dynamics compared to ibrutinib. Whether these experimental differences are clinically relevant remains very difficult to determine. Indeed, the profound functional inhibitory effects of BTKi on neutrophils observed in the experimental setting stand in sharp contrast with what is observed in clinical practice. Invasive aspergillosis remains a rare complication and develops mostly in patients with other risk factors for fungal infections. The reason for these differences is unknown but might be related to experimental conditions that do not recapitulate the complex pathogen-host interactions that occur after conidia inhalation and the role of other cell types such as airway epithelial cells and represent a limitation of the study, together with the limited number of replicates. Thus, one can only partially extrapolate from experimental results the true impact of these molecules on the risk of IFI in vivo. However, these limitations are less a problem since BTKi appear less hazardous in vivo than in vitro.

In conclusion, our results and those of others confirm that all available covalent BTKi are responsible for a plethora of functional defects in neutrophils, irrespective of their kinase inhibition profile. This seems to validate the recent publication concluding BTK is a central component of neutrophils’ antifungal responses [12] and strongly suggests that off-target effects may only play a minor role, if any, in this phenomenon. Why agammaglobulinemia patients are not susceptible to fungal infections remains, however, a very intriguing question. A promising strategy to understand both the role of BTK in neutrophil antifungal activity and the differential inhibitory activity of BTKi will be to evaluate the impact of BTK degraders on neutrophil antifungal activity. BTK degraders belong to a new therapeutic class that targets BTK to proteasomal degradation. BTK degraders specifically disrupt BTK-dependent pathways but have no kinase inhibitory properties hence no off-target effects. Examining the effects of BTK degraders in neutrophils would thus enable the analysis of the role of BTK in antifungal responses in a very specific manner.

## Statement of Ethics

This study was performed in accordance with the Declaration of Helsinki. This human study was approved by Etablissement Français du Sang; Approval No.: ALC/PIL/DIR/AJR/FO/606. All adult participants provided written informed consent to participate in this study.

## Conflict of Interest Statement

DG received honoraria from BeiGene, AstraZeneca, AbbVie, and Janssen and clinical research funding from AbbVie, Incyte, and BeiGene. KL received honoraria from AbbVie, AstraZeneca, BeiGene, and Janssen and clinical research funding from AbbVie and BeiGene. The other authors have no conflict of interest to declare.

## Funding Sources

This work was supported by the Agence Nationale de la Recherche ANR JCJC grant ANR-22-CE15-0041-01 (to B.S.M.). It has also benefitted from the support provided by the University for Advanced Study (USIAS) for a fellowship, within the French national program “Investment for the future” (IdEx-UNISTRA) (to B.S.M.). This study was additionally funded by a grant from BeiGene (to D.G.). The funders had no role in the design, data collection, data analysis, and reporting of this study.

## Author Contributions

D.G., B.S.M., and K.L. designed the study. D.G. and B.S.M. wrote the manuscript and analyzed the results. B.S.M., A.S., T.T., N.B., J.D.F., M.V.B., and T.F. performed the experiments.

## Data Availability

All data generated or analyzed during this study are included in this article and its online supplementary material files. Further inquiries can be directed to the corresponding author.
